# Annual Address before the Erie County Medical Society

**Published:** 1868-02

**Authors:** J. R. Lothrop

**Affiliations:** President


BUFFALO

### Medical and Surgical Journal.

VOL. VII. FEBRUARY, 1868. No. 7.

### Original Communications.

ART. I.—*Annual Address before the Erie County Medical Society.* By J. R. LOTHROP, M. D., *President.*

(Published by vote of the Society.)

It has seemed to me that it will not be inopportune to speak of some things which relate to the public estimation of the profession, and its present position as to its aims, limits, and duties.

The profession has a standing of trust and honor with the public. This is true, even though there are those who doubt its methods, disparage its motives, and would deprive it of its just honors. They wish to bring it into discredit, because through ignorance and prejudice, they hold wrong opinions of its aims and its powers. Even its true friends, while they give it honor, somewhat misjudge it. They do not rightly estimate its functions, or fully appreciate its limits; i. e. they do not form correct notions of just what medicine, as a profession ought to attempt or can accomplish. The physician is too much regarded in his relations to the cure of disease, and therefore as of service only to the sick. He is too little thought of as an adviser to those in health. They are apt to forget that, in his true function, he is a doctor; i. e. teacher of the laws of health, as well as those of disease. Moreover, in his relation to the sick, he is, almost always, expected to effect more, by his medicines, than can be reasonably hoped for, nay, even more than he is willing to promise, however prodigal of promises individuals of his class may be.

This arises from the fact, that there is in the popular mind, a wide-spread belief that diseases are cured only by medicines. This belief, in part well-founded, is in so wide a sense erroneous. Diseases are cured by medicines; no one will hesitate to accept this as a truth; but all diseases are not, neither are they curable by them. Therefore the prevalent feeling that they are curable only by medicines, is without foundation. Yet this belief has had a very important influence upon the practice of medicine. Physicians have often felt compelled to use medicines, governed in doing so, more by the influence arising from this belief, than by any necessity created by the disease. They have not felt free to withhold them, even when to do so would have been the best course, because the feeling has almost always existed, that they were needed.

I do not mean to say that the belief has not had its influence on medical men, and does not now exert it. Without doubt there are many physicians who feel an unwise confidence in the curative powers of medicine. They are not influenced by any feeling of what the public may expect; they use them from a conviction of their necessity and usefulness. Such, however, are not the best minds in the profession. The ablest and wisest physicians know and teach, that medication has its limits, which must be regarded in practice. They feel that the office of the physician is higher and nobler than that of a mere dispenser of drugs, and that he does not cease to be wise and useful, because he does not propose them to the sick.

Whence arose this wide-spread belief in the powers of medicines? Whence came such a notion of disease; as if it were a devouring agent, only to be stayed within the body, by some antagonism brought into it from without? It may be answered, that the profession is largely accountable for it. It has sent it forth, and to its doors must it be brought back. Physicians, in all ages, have sought to inspire confidence in their medicines; partly because they believed in them, and partly because they were moved by the desire to be as helpful as possible. For, it is doubtless true, that benefit comes of having the beliefs of the sick on the side of the means the medical may employ, medicines among others. Hence the teachings of the profession, from the earliest times, have had the effect of fixing in the popular mind, faith in the curative powers of medicines. This belief, then, as it exists to-day, is the growth of centuries, and has become firmly rooted, too firmly to be easily removed. The wrong teaching and wrong learning of two thousand years cannot be corrected in a day. If truth exerts a power by appearing venerable, the same is true of error. It is the more pernicious and difficult to correct that antiquity gives it a false value and currency. The medical mind of to-day is largely emancipated from the old teaching and belief, but they will linger yet a long while in the popular mind, even under the better teaching which the best men of the profession encourage—that better teaching which is the imperative demand of to-day.

I have said that as regards just what he can accomplish by medicine the physician is placed in a false position in public estimation. This I said depends upon a wrong notion of the powers of medicines; a notion almost universal, but originating mainly in erroneous medical teaching. There is another feeling concerning the physician and his medicines, that operates, though in a less degree, to influence public judgment. It is, however, far from being universal, it affects only a portion of the public, and that not the educated or thinking portion. Partial as it is, it yet has its influence. It is but a remnant of old superstitions, which, in the early and rude ages of the world, when diseases were thought to have a supernatural origin, pervaded all minds. We cannot say that now any portion of a modern enlightened public, has the old belief in the power of incantations, charms, or magic to avert or cure disease. This, we trust, has ceased to exist, but there still remains a feeling which borders on it, enough at least to mark its traces. There yet lingers excessive and unwise credulity, a belief in something occult, something which lies just outside of the common experience of the working of natural laws. Hence medicine is much less likely than any other profession to get a candid judgment, and pass for just what it is really worth.

True as this is of medicine itself as a profession, it is even more true of individuals. The estimate of particular men is partial, or exaggerated. One gets more credit than another from the feeling that in some way he has got possession of the hidden things of nature, not by having wrung them from her, through force of mind and study, but by special insight or peculiar endowment. As in times past the superstitious beliefs so ruled minds that something more than simple truth was needed to gain faith, so under this modern feeling, plain truth is not always the most likely to be received. Truth, in her simplicity, has much less the look of reality than ingenious error.

The error underlying such notions is very apparent. The successful practice of medicine is a matter of mental capacity, not of special gifts, or accidental discoveries. One man, more than another, may have a mental aptitude for the physician's work. Doubtless a fitness, more or less peculiar, exists in some men for any one business or calling, above another. When the calling and the man are mutually suited, we see the best achievement. But, except with genius, whose powers are exceptional, this is much less than is often supposed. The physician forms no exception to this general statement. Any man of such force and abilities, as would give him success in other professions, will, with equal effort, secure the same success in the practice of medicine. No one man of good capacities has, above another, the special power to wring from nature a secret which shall fit him to deal with diseases. Good original powers, thorough study and experience, are the physician's best gifts. Therefore the best men ought to secure the grea'est confidence and success. I am not prepared to deny that they do, but still it is true, in a measure, that a belief in the possession of special gifts, or of some secret of nature hidden from most men, has an effect, *small* it may be, and *unjust* it must be, on the popular judgment of a physician's merits.

This must not be taken in too broad a sense. Yet there is in it a partial truth. It has its influence in medical matters to such an extent, that the choice of a physician or some one having the relation of physician, by men of good judgment, is not made with the same sagacity that they show in other affairs of life. In ordinary business they do not place trust, or give credit for ability, without evidence that they are justly due. But for dealing with their diseases they require little or no evidence of fitness for the important work. Confidence is given often to him of whom they know least, and of whose fitness for the important work they entrust to him, they have no proof whatever. When they bring the same common sense to bear upon the treatment of disease that they do upon business affairs, the physician will be more truly judged and more justly honored. For what is the office of the physician, let me say of the well-instructed and honest physician—not every one who undertakes to treat the ills of flesh—but as he is called, the regular physician. This can be best answered by stating the aim of medicine, I may say its practical aim, for it has its scientific side, seeking to prove what is true for truth's sake.

The statement can be briefly made. It is all comprised in this, that it seeks to prevent and cure diseases. Prevention has been less prominent than cure, for the reason that medicine has had more authority in the latter. Its teachings for preventing disease have had much less weight, than its measures for its relief, and very wrongly so, for its office of prevention is equally important. It is as important to know the laws of health as those of disease. This has not happened, because medicine has been untrue to its noblest function. It has happened, because men have chosen rather to persist in unhealthy modes of living, and call upon the medical art to rescue them from the result, than to heed its teachings. They have been much less inclined to be at the trouble to avoid the causes of disease, than to avail themselves of the means of cure. Advice has been much less welcome than medicines. It is not to be expected that medicine can have much weight in private hygiene, till it is tolerated. If men will not be told that they eat and drink improperly, wrong sleep, and construct and beat badly their houses, it must not be charged upon medicine that diseases come. It should not perhaps be broadly stated that in these respects men always consciously err, for there is much ignorance as to what is best, and men ought to be willing to be taught how to gain and keep that health, which once lost, medical treatment may never restore. But it is largely true, that if medicine has been wanting to its highest trust, it has been due to the slight authority which has been given it.

These observations do not bear equally upon public hygiene. In this noble science, medicine has wrought most worthy achievements, and secured its purest and best triumphs. It has lifted up its voice for all sanitary measures likely to promote public health—pure water, drainage, healthy food, pure air, and cleanliness. It has cleared up the mystery which shrouded the source and nature of epidemics, and thereby stayed their fearful march. In public hygiene, however, the medical profession is hardly enough regarded, and public sanitary regulations would be more wisely ordered if physicians were more often consulted.

But however medicine may have failed of the highest good in prevention, in the cure of disease, there has been no lack of the most earnest and careful labor. It has pushed its inquiries throughout all nature. Immense has been its work, and immense have been the results. It is not proper here to enumerate what has been done. It is enough to speak of the spirit and aim of its efforts. Its spirit is as broad and its aim as noble as truth, nothing more or less. It has no theory to propound to which facts must bend. It works in no narrow lines. Whatever experience has shown to be, or whatever promises to be of service in human ailments, all remedies, if really remedies, external or internal, liquid or solid, it brings into its service. It employs drugs in doses large or small, as may be most useful, whatever the theory of their action. In spite of its alleged hostility to anything new, and its proclivity to walk in old ways, I believe it willing to follow the leading of truth, demonstrated truth, let it lead whither it may; even if it should thereby be brought to the doctrine of similarities, or to the absurdities, as they now appear to it, of the "attenuations." But as yet it has no doctrine of similarities nor of contraries, nor of some other method which is neither like, unlike, nor contrary. It is not truly described by any of the "pathies." For it is neither homœopathic, antipathic nor allopathic, or as it is sometimes called heteropathic, however heterodox it may be thought by some. Regular medicine proposes no theory of the action of medicine which shall cover the method of cure. It is difficult enough to ascertain what will cure, without being at the trouble to find out or describe the method according to which it is effected.

We may then say of it, that its chief work and purpose concerns the human body with its complex structures and functions, its health, its diseases and the means of relieving them. Medicine is charged with an obstinate conservatism because it is not ready to adopt whatever is untried, and because while it can only arrive at anything useful by a study of facts, it will not accept as fact everything that has the appearance of it. Medicine is not unwilling to *try* what is new, it is only unwilling to adopt hastily everything new. Its labors have brought out nearly all discoveries that are of any value, at the present time, however much some recent systems may claim. In some branches this is entirely true. All that is known in anatomy, physiology, and pathology, is the results of its labors. In the treatment of diseases, although this is not wholly true, as the proverbial "old woman" must have the credit of some discoveries, it is mainly so. If it has resisted in a few instances the use of remedies which have been proved to be useful, and afterward adopted them, it has resisted and banished thousands of worthless, disgusting, or harmful ones. Its conservatism is a salutary protection to the public, which has received a thousand times more injury from disregard of its unwillingness to adopt the untried, than ever has or ever will be experienced from its unwise or wrong use of its chosen means.

Having said thus much of medicine, commonly, though not very correctly called allopathic medicine, and having made allusions to methods of cure, it appears to me not out of place to speak of homœopathic medicine. This can hardly be avoided in connection with the consideration of the opinions held by the public as to the cure of diseases. It is not to be presumed that the public is interested in systems of medicine, any farther than concerns their success and usefulness. It mainly seeks its own benefit, irrespeetive of the truth or falsity of methods. But inasmuch as homœopathy claims not only more truth, but even a greater success than regular medicine, I deem it not ill-timed to endeavor to speak upon these points, aiming to do so in all fairness and candor. I do not desire that it shall prove to be untrue. I have no wish to detract from its merits or to undervalue its success. If they exist I am willing that they should be taken at their just value. It is undeniable that homœopathy is believed in and adopted by persons in the community who have good sense and judgment in their business affairs and general conduct of life. It seems to them to have merits which are special, and thus commends itself to their choice. That sensibie men and women believe in anything, is a sort of evidence that there is something in it. It is not enough to say that it is a mere delusion. It requires something more tangible than mere imagination to govern the convictions of people of good sense, for any length of time.—What they observe about it, is probably this, that in many cases sick people get well as quickly when treated according to its methods, as by any other methods. Granting that this is a genuine observation—though I think homœopathists will not be content with so small an admission, but claim that the sick in all cases get well *more* quickly—granting I say that their observation is correct; it is easy to understand why they should prefer small doses of medicine. And, theory apart—which probably does not enter into the thoughts of many who call upon homœopathy—that in it, which most affects them is, that its medicines are not hard to take. In other words their preference is almost wholly governed by its small doses.

Homœopathy is founded upon a theory of the action of medicines. Its distinctive feature is the doctrine of similarities, or as commonly stated, like cures like. According to this doctrine medicines in order to cure must cause symptoms similar to those to be removed; or to state it in other words, medicines which cause certain symptoms will cure those symptoms when they appear. In order to cure headache you must apply a medicine which will cause it, even cause more headache; for Hahnemann taught that a stronger artificial disease must be created by medicine to remove the natural one, and then an end could be put to the artificial disease by taking away the medicine which caused and kept it up, leaving the body in health. It is largely a treatment of symptoms, and has little to do with causes; yet it must attempt to treat of causes, as no system which ignores them can long be worth notice. Added to this is the doctrine of infinitesimal doses. Homœopathy is a system of practice, and does attempt anything beyond the treatment of diseases by medicine. It has no different system of anatomy, physiology, or pathology. It takes them as taught and improved by regular medicine. It adds nothing to them. It has contributed no facts to either, nor shed any light upon the nature of diseases. Its claims rest upon its theory, its medicines, and its doses. Take away its materia medica, and it would be left with nothing but its doctrine. Men get a good deal of comfort out of a sound doctrine, provided its soundness is beyond question. To establish the doctrine there is a sort of appeal to facts in their "provings," which, it must be granted, might prove something, if conducted fairly and upon a large scale. For if a symptom is constant in a large number of provings, it has a fair claim to pass as a fact. But there are no provings made with the requisite care and precaution, and in sufficient numbers, to make them of any value. Many of the recorded provings are trivial, absurd, and even indecent in their enumeration of symptoms.

But however much the argument may run against its theory and its practice, it is sufficient to its friends, that they can believe it to be successful. Its *success* is its vital point, and so its friends feel. For if it is not successful it cannot hope to endure long. Nothing but success could carry along a doctrine and practice which in many respects contradict common experience, and which to the plainest common sense, in other respects, seem to border on absurdity. Accordingly a great deal more is said of its wonderful success, than of the truth of its doctrines.

It must be admitted that many who are treated by it get well. This is not so very wonderful when we observe that under all kinds of treatment, perhaps we may say in spite of all kinds of treatment, sick people get well. But that they do so more speedily, or more certainly under it, there are no facts in proof. That there is a belief of this sort need not surprise us, when we remember the history of medical delusions, and of all kinds of quackery. The most absurd measures for cure have hardly been too absurd to prevent their getting the faith of some adherents.

That its advocates believe they have facts is true, but their attempts to draw inferences from experience must fail, because they do not and cannot know how to interpret experience. No one but a student of the physical sciences can understand the great precaution needed to get at a fact, and therefore the common experience of life does not fit men to judge correctly of what is actually true in such matters.

The argument against homœopathy rests, first against the theory. The doctrine of similarities is not a truth. At best, if there is anything in it, it is but partially true, and therefore is a poor foundation for a system of treatment. It is, however, but a truth in appearance, not in reality; a seeming, not a real truth. However some instances may be brought forward which seem to confirm the doctrine, the doctrine itself is entirely baseless, as much so as the old doctrine of signatures from which it appears to have arisen. The old fanciful doctrine of signatures was based upon resemblances in the medicines themselves, and not in the symptoms they caused to the organ they were to affect, and many of the remedies employed according to it, are now included in the homœopathic materia medica, and for the same uses. These resemblances related to form, color, quality, etc. When we read of burnt foxes' tales as a remedy for asthma, beeause the fox is long. winded, we are reminded of some of the fancies of the doctrine of similarities. It is not true that diseases are cured by creating an artificial disease with similar symptoms, any more than that bryony cures dropsy beeause its root has the shape of the swollen foot of dropsy; or that the little plant, eye-bright, cures diseases of the eye because it has in the centre of its flower a black spot resembling the pupil of the eye. Yet this absurdity had once believers. It certainly was a sort of doctrine of like cures like, and was probably not without its influence on this modern one.

In the second place, the doctrine of infinitesimal doses seems too much of an absurdity to have been proposed or believed by any reasonable being. Imagine the mental status of a man, in sound health, who could sit down under the influence of a decillionth of a drop of laudanum, or of a grain of charcoal to prove its effects and note its symptoms. Much more of a sick man who could have faith in the efficacy of such a dose, though often repeated. But even if we are compelled to admit, as is claimed, that however absurd it may seem, it is possible that there are positive effects from such a dose, it certainly is contrary to common experience in all other matters, that the potency of medicine is increased by lessening the dose, so that, in a sense, the less a man takes the more powerful is the effect.

Of about the same value is the oft-repeated saying that their medicines can do no harm if they do no good. First, because the dose is small, and secondly because being given to remove one disease by causing another, if in any way they fail of this, they have no bad effect. If a medicine is powerful at all, it will do harm in proportion to the dose. The less the dose, the less the harm. It is asserted that in small doses medicines are even more powerful for good than large; why not then for harm? But many of our best medicines have a power of harm proportioned to their capacity for good, the one being almost a measure of the other.

To give a medicine then that can do no harm, is equivalent to giving one that can do no good. The mere negative of doing no harm is a poor recommendation.

But the greatest argument against the system is, after all—I believe the assertion is not untrue or unfair—that it is not adhered to in practice. Medicines are given by homœopathists upon the theory of contraries, as much as upon that of similarities, and in sensible doses; and consequently there can be no honesty in a practice which claims to be very different from, even antagonistic to, regular practice, aud yet is not. Homœopathy if it is not true to itself, has no peculiar merits, it is simply giving medicines as given by the regular profession, in small doses. It would not be difficult for us to admit that medicines in small doses are often quite as good and perhaps better than in larger ones.

I am not going to deny that homœopathy has, apparently, had some influence on the course of regular medical practice. The belief on the part of the public that less medicine was equally efficacious, has had upon the whole the result to lessen the amount. It would be useless to deny that there has been over-medication, and let homœopathy claim its share, whatever it may be, in lessening it. But it must not claim too much, for the progressive tendency of medicine has had a thousand fold more agency in producing it. It has yet a high work to do, viz: to teach that in many diseases not less medicine is needed, but that the result is as good without any, and thus it will be more honest and more useful than homœopathy could be if true to itself; satisfying the belief that medicines are needed, by an appearance only of giving them, and thus keeping the promise to the ear and breaking it to the sense.

And now to learn this part of the subject, I think I may say that, setting aside its theory which is at least only partially true, if it is honest it is simply appearing to give medicine, while people get well; or if dishonest, it is an unworthy way of influencing people into a hostility to regular medicine while it uses its remedies and experience. If it has been of service to humanity let us give it honor, if it has any basis of truth it will endure, we need not fear to speak candidly of it, lest we help it. Speaking as fairly as is possible of what one feels to be a delusion, it seems to me it can only rank as a pseudo-science, not having even the merit of discovery, as it seems certain that its essential dogma is to be found in the old and fanciful doctrine of signatures. No system of practice founded upon a mere theory ever has been long-lived, nor will any so founded probably long endure; while regular medicine has survived centuries, accumulating wisdom, aiming at progress, seeking earnestly for truth, and enriched by the lives and labors of pure, able, and noble men.

Having spoken of the relation of the profession to the public, it remains now to say a few words of the profession itself, and our duties to it. The profession imposes upon us duties. It is our duty to increase its honor and estimation among men, and to fitly and thoroughly educate ourselves for its work.

Our first duty to the profession is to present truthfully its claims to public confidence. Not claiming for it more than it deserves, but always, candidly and fairly stating its powers and its limits. What is its position to-day? As far as it is a science, one of progress and promise. As far as it an art, imperfect, without laws, and even destitute in a great measure of clearly-ascertained, well-established facts. Yet the medical art of to-day is a vast gain upon that of the past. Clinical experience, the microscope, and autopsies are every day more clearly teaching what can and what cannot be done; or at least what it is wise to attempt and what not. Our measures are not as purely empirical. Though we have no laws of therapeutics, we are more definite in our aims, and we can count upon more certainty in our results.

But we should state fairly its limits, for we are well aware that they exist. No duty is more imperative than to correct the erroneous notions held by the public as to the powers of medicines. We must teach that medicines in many cases do not cure diseases, and are therefore not needed; nay, are even perturbative.

In spite of all fears that may be felt lest harm may come to our art in the loss of confidence, truth and honesty demand it. A profession which deals honestly with the public must inevitably secure its trust. Homœopathy thrives to-day in consequence of the belief that medicines are always necessary for the cure of diseases. They have medicines for every symptom which may be felt or imagined, and they are to-day the most thorough advocates of the necessity of medicines in diseases; and in that respect fall in with the popular belief, or seem to do so. As far as the public is concerned, it matters not that their doses or medicines are inert, as long as it satisfies the belief and answers to the desire for dosing. It cannot be worse for regular medicine to honestly declare that in many diseases medicines are not needed. For once let the belief become fixed, and men will be better satisfied to take no medicine, than to take even the most minute doses of it. However distant it may be, we may look forward to the time when the advice of an honest and enlightened physician will be of as much value as his medicine, and when reasonable men will be convinced that a cure can be affected as quickly by the former as the latter. Men do not take medicine, even in infinitesimal doses for any pleasure that it gives them, but from a belief that it does them good. Remove this belief and they will not take it for an amusement. They will not, as now is often the case, take small globules, or even bread pills every day in the year, for real or imaginary diseases, and be about the same at the end as at the beginning.

Let it be borne in mind that I am not discrediting medicines. I believe in their benefits. Many of them have directly brought health and solace to humanity. They are most distinctly curative in many diseases. Their proper use annuls pain, controls disease, and gives new hope to man. If their use is restricted to cases needing them, they will occupy their proper place, have honor and escape reproach.

Our duty to our profession demands a higher and broader standard of education. We need minds trained to a wider range of study, in order to get the best progress in medicine. A narrow and untrained mind is incapable of making the most of its experience. In order that observations may lead to correct conclusions, the mind must be kept right by a habit of sound induction, and correct inferences from facts. In a study like that of medicine in which established facts are hard to be found, and in which rules are almost impossible, there is a necessity for the best mental training to save from theories and hasty generalization. The need of the time will, perhaps, have the result to bring about the fulfillment. But as a profession we should make our demands for a better education. The movement in our colleges in that direction is a salutary one. It is not contended that for professional success in the best sense, i. e. a success which is founded upon making the best use of what is known in medicine—that the education should be what is called liberal. It need not embrace classical study. But it should be such that a physician is not only capable of understanding the common physical facts of the world in which he lives, but should be also capable of appreciating the best thought and knowledge of the time. He certainly ought to have such a knowledge of elementary scientific truths, beyond his special science, as shall enable him, if not to speak with authority himself, to know what is genuine science and what is not.

I hold, that the broader a man's culture, the better he can read such facts as his experience gives him, the sounder will be his conclusions, and the more useful and honorable will be his career as a physician; to say nothing of the elevation and dignity it will impart to his profession. It may be said that as a science medicine is extensive enough to occupy all the time and thought which is not taken up in practical work. But this is not so. It is possible to combine a thorough knowledge of all that pertains to medicine, with a great deal of general knowledge of other subjects. If it were true that a man could know but one thing well, or to know it well he must neglect all others, we might well feel that progress was endangered. Continue the argument a little further and you might confine study to a single branch of a single science.—Already specialists in medicine are advancing this idea. They say it is not possible to know everything, therefore know one thing well. Were the idea carried out, and medicine divided up among specialists, it would be destructive of any large views, and physicians would become the most narrow, and except in one direction, the most ignorant of educated men. For all purposes for which a man of breadth and culture is worth something to the world, he would be of little value. He would live in his own narrow sphere, and in the midst of his own narrow prejudices, useful to that extent, but of little service to human interests in any other. A man may confine himself to one branch or specialty in medicine, in practice, and do his particular thing better than any one else, but if the pursuit of one branch limits his study of all, and excludes general knowledge of many other things, the particular excellence is attained at too great a cost of advancing small things at the expense of the greater. It ought to be, it must be, one of the aims of the profession to insist upon an education which shall not only make its own particular science as well known as it can be, but give some knowledge of other subjects, which concern the world and human beings, and thus make itself capable of enlarged views of its own work and its relation to the public.

Finally, Mill says, "the most incessant occupation of the human intellect, throughout life, is the ascertainment of truth." The physician, as much as any investigator of the truths of science, is interested, and needs to know what is actually true. Besides, that in the dark paths he must go, it is hard to ascertain, he urgently needs it, for upon a knowledge of it depends so much which may be of incalculable benefit to human beings. He, above all men, is interested not to shut his mind against it. And by so much as he is in need of it, by so much should he adhere to and declare it; declare it in reference to his art, which is yet limited nd imperfect; in reference to his own knowledge and what it is possibl to know; in reference to what is best in his dealings with the well and the sick. Regular medicine can afford to be truthful, for if there is much yet that is uncertain, and still more unknown, it cannot charge itself with lack of study or labor to comprehend the unknown, and reads the secrets of nature. If a physician knows all that medicine, up to this time, is in possession of, he need not be unwilling to confess that there are some things he does not know. He need feel this reluctance only when he has failed to possess himself of the knowledge there is. When this spirit actuates the profession, its members will feel the poorness of mere personal aims; and point all aspirations and energies in an unselfish direction. It will give them a new and higher notion of what constitutes success, and will lead them to a reward, which is the best that an unselfish man can desire, viz: that which follows noble aims and endeavors.

---

Blood-Corpuscles in Chlorosis.—M. Duncan, of St. Petersburgh, has just pointed out the remarkable fact that the blood-discs of chlorotic persons yield up their coloring matter more easily than do those of healthy subjects.

